# Analysis of inferior nasal turbinate width and concha bullosa in subjects with nasal septum deviation: a cone beam tomography study

**DOI:** 10.1186/s12903-021-01576-2

**Published:** 2021-04-24

**Authors:** Shishir Ram Shetty, Saad Wahby Al Bayatti, Natheer Hashim Al-Rawi, Hesham Marei, Sesha Reddy, Hossam Abdelatty Abdelmagyd, Sangeetha Narasimhan, Sausan Al Kawas, Asok Mathew

**Affiliations:** 1 College of Dental Medicine, University of Sharjah, Sharjah, United Arab Emirates; 2 College of Dentistry, Gulf Medical University, Ajman, United Arab Emirates; 3 University of Science and Technology of Fujairah, Fujairah, United Arab Emirates; 4 Department of Oral and Craniofacial Health Sciences, College of Dental Medicine, University of Sharjah, Sharjah, United Arab Emirates

**Keywords:** Nasal septal deviation, Compensatory hypertrophy, Inferior turbinate, Cone beam computed tomography

## Abstract

**Background:**

In individuals with nasal septal deviation (NSD), compensatory hypertrophy of the nasal turbinates occurs as a protective mechanism of the nasal passage from dry and cold air. NSD associated nasal turbinate hypertrophy is usually recurrent, requiring repetitive imaging. Therefore, a multiplanar imaging modality with a low radiation dose is best suited for long-term follow-up of this condition. This study aimed to evaluate the association of width of inferior turbinates and presence of concha bullosa with the degree of NSD using Cone beam computed tomography (CT).

**Methods:**

The CBCT scans of 100 patients with NSD were selected as per convenience sampling and were evaluated by two maxillofacial radiologists. The width of the non-hypertrophied inferior turbinate (NHT) on the convex side of the NSD, and hypertrophic inferior turbinates (HT) on the concave side of the NSD were measured at three locations. The septal deviation angle (SDA) and the presence of concha bullosa (CB) were determined.

**Results:**

A significant difference was observed in the anterior, middle, posterior, and mean widths between HT and NHT (*p* < 0.001). There was a significant difference in the widths of the HT and NHT among different types of NSD. A strong positive correlation (r = 0.71, *p* < 0.001) was found between SDA and the mean width of the HT. Age (*P* = 0.71) and gender (*P* = 0.65) had no significant difference among different types of NSD. Regression analysis revealed that the presence of CB (*p* = 0.01) and middle width of the HT (*p* < 0.001) are significant predictors of SDA and type of NSD.

**Conclusion:**

The results of the present study reveal that the middle width of the HT and the presence of CB influence the degree of NSD. The present study results recommend the use of CBCT as a substitutive low radiation dose imaging modality for evaluation of NSD, CB, and associated inferior turbinate hypertrophy.

## Introduction

Nasal obstruction is one of the commonly reported symptoms in clinical practice [1]. It is estimated that 42% of the population may have some form of a nasal septal deviation (NSD) and associated compensatory hypertrophy of the nasal turbinate [2]. The hypertrophy of the inferior nasal turbinate on the concave side of the NSD is called “compensatory hypertrophy” [3]. The main function of this compensatory hypertrophy is to protect the nasal airway passage from cold and dry air [4]. On most occasions, hypertrophy of the inferior nasal turbinate is reversible, however compensatory hypertrophy of the turbinate associated with NSDs is usually persistent [5]. It is also important to note that, if the hypertrophy of the turbinates is associated with NSD there is a thickness in the bony and soft tissue components of the turbinates. Conversely, only soft tissue thickening of the turbinates is observed when any other cause is associated [6]. There are only a few studies that have evaluated the association between the NSD and hypertrophy of the inferior nasal turbinate using computed tomography [7, 8]. Concha bullosa (CB) caused by the pneumatization of the middle turbinate, may occasionally cause nasal obstruction [9]. Although there is an association between NSD and the presence of CB, the cause-effect relationship between NSD and CB is still not very clear [9].

Although studies have investigated incidence of CB and HT in NSD, no studies are investigating the association or influence of these findings on NSD [9]. Our study was conducted to evaluate the association between the width of HT, CB, and NSD. Further, we also attempted to determine the influence of these variables on the type of NSD using CBCT.

## Material and methods

The ethical approval for the study was obtained from the Institutional Review Board, Gulf Medical University. CBCT scans of 100 subjects with NSDs who had earlier reported to the university teaching hospital for various dental treatments, were selected for the study based on convenience sampling.

CBCT scans of C-shaped septal deviation were evaluated by two maxillofacial radiologists. Whenever there was a disagreement a third maxillofacial radiologist was consulted. All the maxillofacial radiologists had 10 years of clinical experience.

The CBCT scans were obtained using Planmeca ProMax 3D machine. The machine functioned at 90 kVp and 10 mA for image acquisition. The maxillofacial radiologists used 9 X 16 cm FOV.

Inclusion criteria: CBCT scans of subjects with anteroposterior C-shaped septal deviation, observed during the radiographic examination were included in the study. Scans of patients between 18 to 75 years of age were included in the study. Exclusion criteria: Scans with errors, artifacts, and incomplete coverage of the area of interest were excluded. CBCT scans of patients with orofacial syndromes affecting skeletal structures and patients with cleft palate were excluded.

The width of the hypertrophied turbinate (HT) and width of the non-hypertrophic turbinate bone (NHT), were measured at the anterior, middle, and posterior thirds of the inferior turbinate in coronal CBCT sections.

**Fig. 1:**
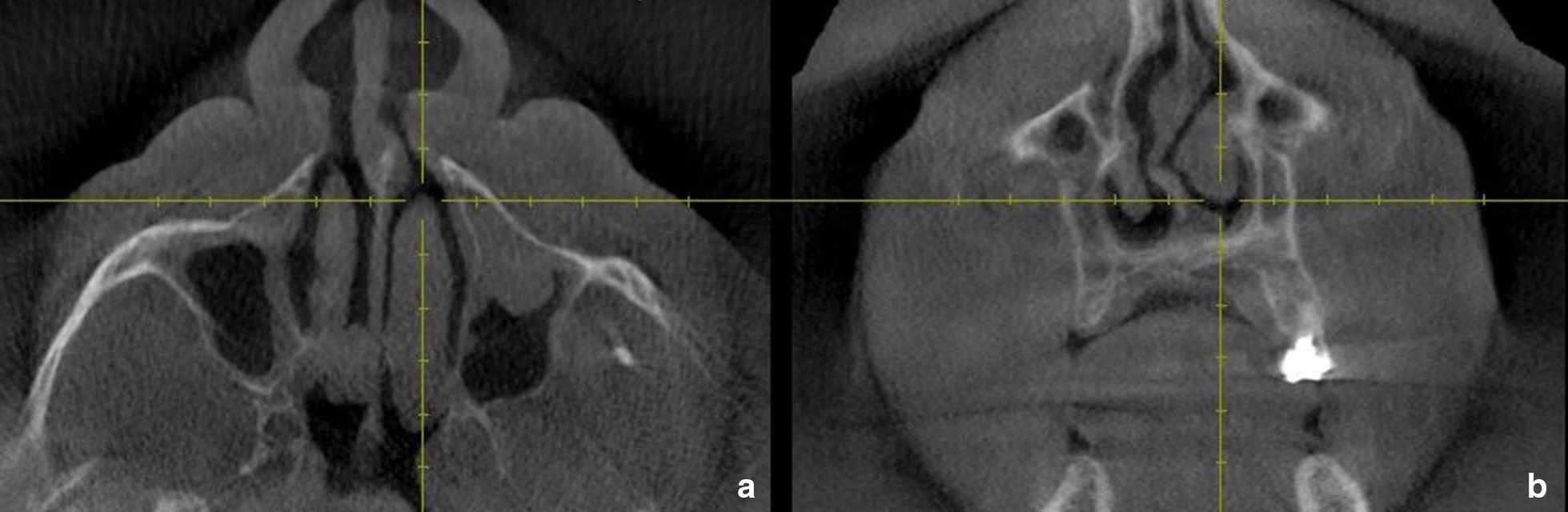
Axial (**a**) and Coronal (**b**) CBCT sections showing the site at which the anterior width of the inferior turbinate was determined in the study

**Fig. 2:**
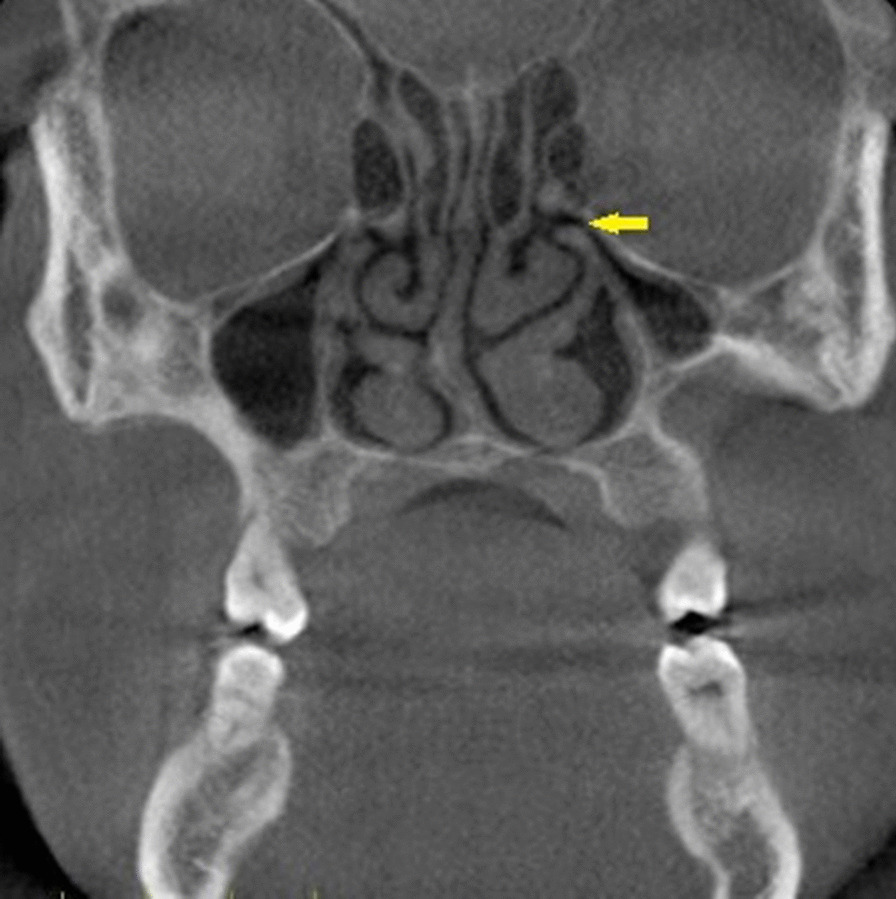
Coronal CBCT section showing the site (ostium and uncinated process) at which the middle width of the turbinate was determined

**Fig. 3:**
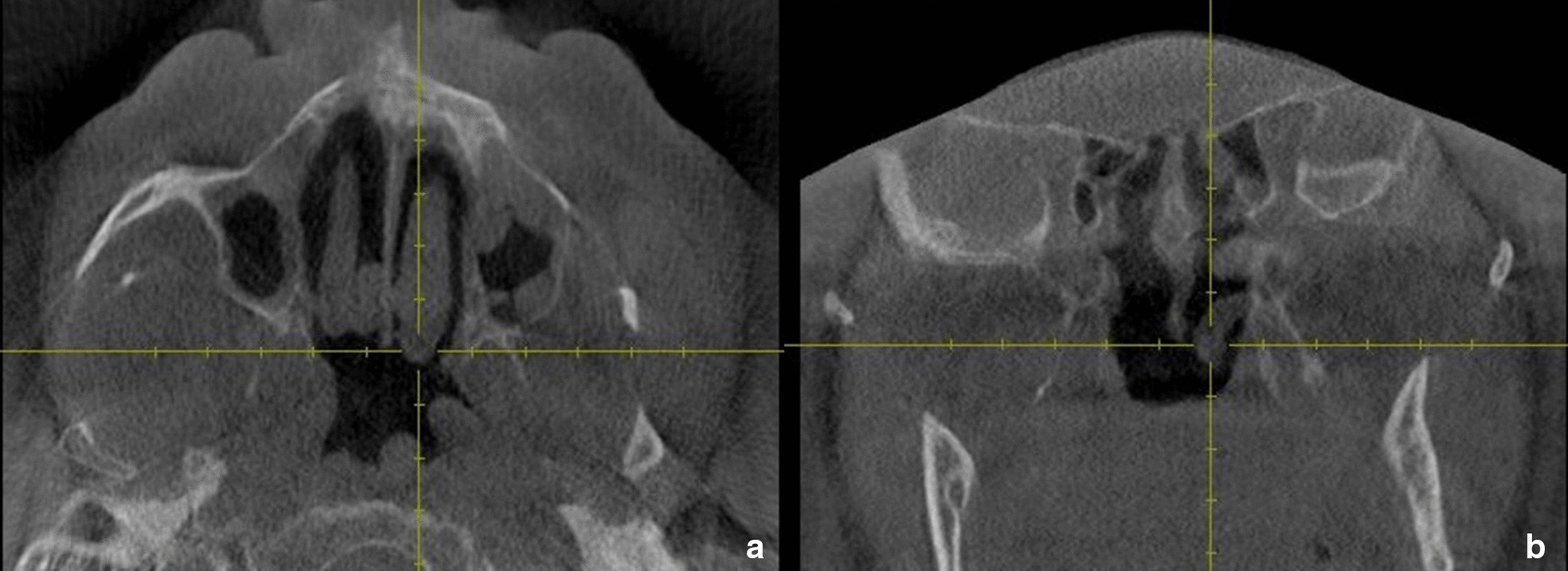
Axial (**a**) and Coronal (**b**) CBCT sections showing the site at which the posterior width of the inferior turbinate was determined in the study

To achieve uniformity of calibration among the evaluators' anterior width was measured on the first image in which the entire inferior turbinate could be identified (Fig. 1a and b). The middle width was measured on the section in which the uncinate process and maxillary sinus ostium were visualized (Fig. 2). The posterior width was measured on the last image in which the entire inferior turbinate could be visualized (Fig. 3a and b) [10].

**Fig. 4:**
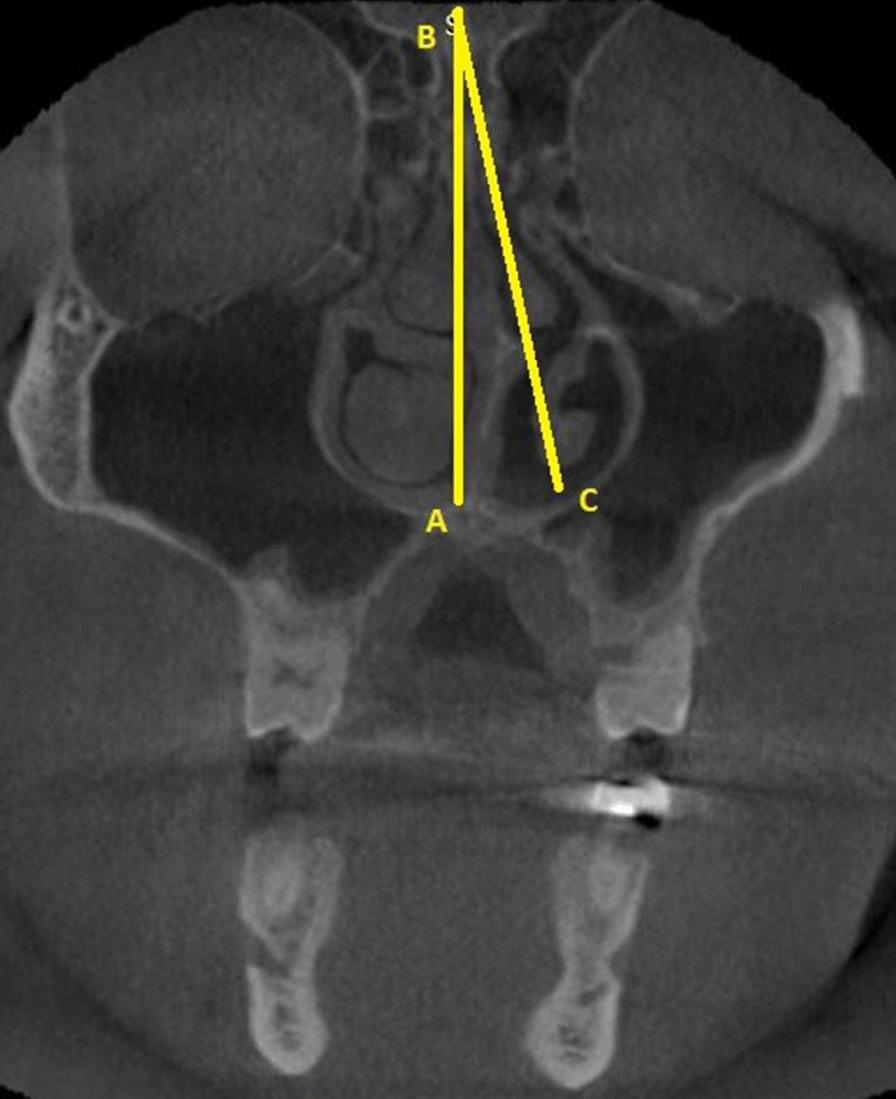
Coronal CBCT section showing method used for calculating septal deviation angle SDA

Only Contralateral CB with a size exceeding 50% height of the middle turbinate was considered [9]. The septal deviation angle (SDA) was measured in the coronal CBCT sections using the criteria by Orhan et al. [7]. The anatomical landmarks used for measurement of the SDA are described in Fig. 4. Point A is the junction of the nasal septum with the floor of the nasal cavity. Point B is the Crista Galli. Line BC is the tangent arising from point B and passing through the outermost part on the convexity of the deviated septum. Angle ABC is the septal deviation angle (SDA). Based on the angle of deviation, the NSDs were classified into mild (less than 9°), moderate (9°-15°), and severe (greater than 15°). This classification was according to the criteria followed by Al-Rawi et al. [11].

The data gained after the evaluation of CBCT scans were statistically analyzed using IBM SPSS statistics (Version 22, Armonk. NY: IBM Corp).

## Results

Cohens Kappa test was used to determine inter-rater reliability 0.84. The Kappa rating rubrics suggested by Regier et al. was applied [12]. The mean SDA of the study subjects was 12.03 degrees. Mild, moderate, and severe types of NSD were observed in 38, 24, and 38 study subjects respectively. When the mean age of the subjects with different types of NSD was compared using ANOVA, no significant difference was observed (*P* = 0.71) (Table 1). Further pairwise comparison using the Tukey Post Hoc test revealed, no significant difference (Table 2).

**Table 1:** Comparison of mean age of study subjects with different types of NSD

	Group	N	Mean	SD	Min	Max	ANOVA	
							F	*p*-value
Age	Mild	38	40.29	12.92	22.00	67.00	0.35	0.71 (NS)
	Moderate	24	43.17	14.64	18.00	70.00		
	Severe	38	41.74	12.86	22.00	75.00		

^*^p < 0.05 Statistically Significant, *p* > 0.05 Non Significant

**Table 2:** Pairwise comparison of the mean age of study subjects between different types of NSD

Dependent Variable	(I) Type	(J) Type	Mean Difference (I-J)	Std. Error	*p*-value	95% Confidence Interval	
						Lower Bound	Upper Bound
Age	Mild	Moderate	− 2.88	3.47	0.69 (NS)	− 11.15	5.39
		Severe	− 1.45	3.06	0.88 (NS)	− 8.72	5.83
	Moderate	Severe	1.43	3.47	0.91 (NS)	− 6.84	9.70

Tukey Post Hoc Test; *p < 0.05 Statistically Significant, *p* > 0.05 Non Significant

When the occurrence of the type of NSD between the genders was evaluated using Chi Square test, no significant difference was observed (Table 3).

**Table 3:** Comparison of gender and the types of NSD

		Type			Total	Chi-Square test	
		Mild	Moderate	severe		Chi-Square value	*p*-value
Gender	Male	20	14	24	58	0.87	0.65 (NS)
		52.6%	58.3%	63.2%	58.0%		
	Female	18	10	14	42		
		47.4%	41.7%	36.8%	42.0%		

^*^p < 0.05 Statistically Significant, *p* > 0.05 Non Significant, NS

When the anterior, middle, posterior, and mean widths of NHT and HT were compared, using the t-test, a significant difference was observed (Table 4).

**Table 4:** Comparison of the anterior, middle and posterior widths between NHT and HT

	Width	N	Mean	SD	Mean Difference	95% Confidence Interval of the Difference		t	df	*p*-value
						Lower	Upper			
Anterior Width	NHT	100	7.78	0.99	− 4.18	− 4.87	− 3.50	− 12.15	99	< 0.001*
	HT	100	11.97	3.92						
Middle Width	NHT	100	8.04	1.18	− 4.13	− 4.75	− 3.50	− 13.02	99	< 0.001*
	HT	100	12.17	3.89						
Posterior Width	NHT	100	6.08	0.91	− 1.38	− 1.81	− 0.94	− 6.28	99	< 0.001*
	HT	100	7.46	2.57						
Mean Width	NHT	100	7.30	0.91	− 3.23	− 3.79	− 2.67	− 11.46	99	< 0.001*
	HT	100	10.53	3.36						

^*^p < 0.05 Statistically Significant, *p* > 0.05 Non Significant, NS

When the anterior, middle, posterior and mean width of NHT were compared among the study subjects with mild, moderate and severe types of NSD there was a significant difference (*P* < 0.001) (Table 5). When a pairwise comparison among the groups was carried out using the Tukey Post Hoc Test, it was observed that there was no significant difference (*p* = 0.27) in the mean width of the NHT between mild and moderate types of NSD (Table 6). However, there was a significant difference in the mean width of NHT between the mild and severe types of NSD. Similarly, there was a significant difference in the mean width of the NSD between moderate and severe types of NSD. The anterior middle and posterior width also showed changes similar to the mean width.

**Table 5:** Comparison of the width of NHT width among the types of NSD

NHT	Type of NSD	N	Mean	SD	Min	Max	ANOVA	
							F	*p*-value
Anterior Width	Mild	38	7.18	0.72	5.23	8.89	27.88	< 0.001*
	Moderate	24	7.58	0.82	6.06	9.45		
	Severe	38	8.52	0.84	6.67	10.99		
Middle Width	Mild	38	7.25	0.67	5.65	8.45	37.87	< 0.001*
	Moderate	24	7.77	0.67	6.43	8.67		
	Severe	38	9.00	1.17	7.45	11.33		
Posterior Width	Mild	38	5.80	0.76	4.01	7.88	12.02	< 0.001*
	Moderate	24	5.72	0.68	4.88	7.56		
	Severe	38	6.60	0.94	4.67	8.45		
Mean Width	Mild	38	6.74	0.63	5.33	8.08	35.20	< 0.001*
	Moderate	24	7.03	0.58	6.11	8.33		
	Severe	38	8.04	0.82	6.59	9.48		

^*^p < 0.05 Statistically Significant, *p* > 0.05 Non Significant, NS

**Table 6:** Pairwise comparison of the width of NHT between individual types of NSD

TNHT	(I) Type	(J) Type	Mean Difference (I-J)	Std. Error	*p*-value	95% Confidence Interval	
						Lower Bound	Upper Bound
Anterior Width	Mild	Moderate	− 0.40	0.21	0.13 (NS)	− 0.90	0.09
		Severe	− 1.34	0.18	< 0.001*	− 1.77	− 0.90
	Moderate	Severe	− 0.93	0.21	< 0.001*	− 1.43	− 0.44
Middle Width	Mild	Moderate	− 0.52	0.23	0.07 (NS)	− 1.07	0.03
		Severe	− 1.75	0.20	< 0.001*	− 2.24	− 1.26
	Moderate	Severe	− 1.23	0.23	< 0.001*	− 1.78	− 0.67
Posterior Width	Mild	Moderate	0.08	0.21	0.93 (NS)	− 0.43	0.59
		Severe	− 0.80	0.19	< 0.001*	− 1.25	− 0.35
	Moderate	Severe	− 0.87	0.21	< 0.001*	− 1.38	− 0.36
Mean Width	Mild	Moderate	− 0.28	0.18	0.27 (NS)	− 0.71	0.15
		Severe	− 1.29	0.16	< 0.001*	− 1.67	− 0.91
	Moderate	Severe	− 1.01	0.18	< 0.001*	− 1.44	− 0.58

Tukey Post Hoc Test *p < 0.05 Statistically Significant, *p* > 0.05 Non Significant, NS

When the anterior, middle, posterior, and mean width of HT were compared among mild, moderate and severe types of NSD there was a significant difference (*P* < 0.001) (Table 7). When a pairwise comparison among the groups was carried out using the Tukey Post Hoc Test, it was observed that there was a significant difference in the anterior, middle, posterior, and mean width of the HT among mild, moderate and severe types of NSD (Table 8).

**Table 7:** Comparison of the width of HT among the types of NSD

HT	Type	N	Mean	SD	Min	Max	ANOVA	
							F	*p*-value
Anterior Width	Mild	38	9.42	2.18	5.34	14.78	28.16	< 0.001*
	Moderate	24	11.50	3.22	7.08	18.11		
	Severe	38	14.81	3.85	8.08	20.11		
Middle Width	Mild	38	9.64	2.00	6.74	14.45	29.83	< 0.001*
	Moderate	24	11.56	3.23	8.11	18.56		
	Severe	38	15.07	3.83	9.04	20.28		
Posterior Width	Mild	38	6.27	1.19	4.01	9.76	14.71	< 0.001*
	Moderate	24	6.89	2.44	4.88	12.34		
	Severe	38	9.00	2.90	5.11	15.09		
Mean Width	Mild	38	8.45	1.65	6.26	12.37	26.77	< 0.001*
	Moderate	24	9.98	2.85	6.98	16.34		
	Severe	38	12.96	3.41	8.04	17.33		

^*^p < 0.05 Statistically Significant, *p* > 0.05 Non Significant, NS

**Table 8:** Pairwise comparison of the width HT between the individual types of NSD

HT	(I) Type	(J) Type	Mean Difference (I-J)	Std. Error	*p*-value	95% Confidence Interval	
						Lower Bound	Upper Bound
Anterior Width	Mild	Moderate	− 2.08	0.82	0.03*	− 4.04	− 0.13
		Severe	− 5.39	0.72	< 0.001*	− 7.11	− 3.67
	Moderate	Severe	− 3.30	0.82	< 0.001*	− 5.26	− 1.35
Middle Width	Mild	Moderate	− 1.91	0.81	0.04*	− 4.83	− 0.11
		Severe	− 5.43	0.71	< 0.001*	− 7.12	− 3.74
	Moderate	Severe	− 3.52	0.81	< 0.001*	− 5.44	− 1.59
Posterior Width	Mild	Moderate	− 0.62	0.59	0.01*	− 2.03	0.79
		Severe	− 2.73	0.52	< 0.001*	− 3.97	− 1.49
	Moderate	Severe	− 2.11	0.59	0.002*	− 3.52	− 0.70
Mean Width	Mild	Moderate	− 1.54	0.71	0.04*	− 4.23	0.15
		Severe	− 4.52	0.62	< 0.001*	− 6.00	− 3.03
	Moderate	Severe	− 2.98	0.71	< 0.001*	− 4.67	− 1.29

Tukey Post Hoc Test *p < 0.05 Statistically Significant, *p* > 0.05 Non Significant, NS

When the width of the NHT and HT were correlated with SDA there was a significant correlation (*P* < 0.001), The width of HT showed a strong correlation with SDA whereas the width of the NHT showed moderate correlation with the SDA (Table 9).

**Table 9:** Correlation between the width of NHT and HT with SDA

	Region	SDA	
		Correlation Coefficient	*p*-value
HT	Anterior Width	0.68	< 0.001*
	Middle Width	0.70	< 0.001*
	Posterior Width	0.76	< 0.001*
	Mean Width	0.71	< 0.001*
NHT	Anterior Width	0.52	< 0.001*
	Middle Width	0.57	< 0.001*
	Posterior Width	0.56	< 0.001*
	Mean Width	0.55	< 0.001*

^*^p < 0.05 Statistically Significant, *p* > 0.05 Non Significant, NS

When the width of HT and NHT was compared in between study subjects with and without concha bullosa a significant difference was noted (*p* < 0.001) (Table 10). Similarly, when the SDA of study subjects with and without concha bullosa was compared a significant difference was observed (*p* < 0.001) (Table 10).

**Table 10:** Comparison of the width of NHT, HT and SDA according to the presence of concha bullosa

		Concha bullosa	N	Mean	SD	Mean Difference	95% Confidence Interval of the Difference		t	df	*p*-value
							Lower	Upper			
NHT	Anterior Width	Absent	63	7.53	0.87	− 0.68	− 1.06	− 0.29	− 3.49	98	0.04*
		Present	37	8.21	1.04						
	Middle Width	Absent	63	7.72	0.98	− 0.87	− 1.33	− 0.42	− 3.81	98	0.02*
		Present	37	8.59	1.30						
	Posterior Width	Absent	63	5.93	0.84	− 0.42	− 0.78	− 0.05	− 2.26	98	0.03*
		Present	37	6.35	0.97						
	Mean Width	Absent	63	7.06	0.79	− 0.65	− 1.01	− 0.30	− 3.71	98	0.04*
		Present	37	7.72	0.96						
HT	Anterior Width	Absent	63	10.85	3.21	− 3.02	− 4.52	− 1.52	− 3.99	98	< 0.001*
		Present	37	13.87	4.32						
	Middle Width	Absent	63	11.06	3.17	− 3.00	− 4.49	− 1.50	− 3.99	98	< 0.001*
		Present	37	14.05	4.30						
	Posterior Width	Absent	63	6.91	2.09	− 1.48	− 2.50	− 0.46	− 2.89	98	0.005*
		Present	37	8.39	3.03						
	Mean Width	Absent	63	9.61	2.74	− 2.50	− 3.79	− 1.21	− 3.84	98	< 0.001*
		Present	37	12.11	3.75						
	SDA	Absent	63	10.13	5.08	− 4.96	− 6.92	− 3.00	− 5.03	98	< 0.001*
		Present	37	15.09	4.15						

^*^p < 0.05 Statistically Significant, *p* > 0.05 Non Significant, NS

Linear regression to predict SDA based on study variables revealed that the presence of concha bullosa and middle width of the HT are significant predictors of SDA. The presence of concha corresponds to a 2.10 unit change in the SDA (R2 = 0.19) and for every unit increase in middle width of the HT, there is a 2.28 unit change in SDA (R2 = 0.42). The presence of CB was associated with a significant increase in the width of HT and NHT. (Table 11).

**Table 11:** Linear regression to predict SDA based on study variables

	Unstandardized Coefficients		Standardized Coefficients	t	*p*-value	95.0% Confidence Interval for B	
	B	Std. Error	Beta			Lower Bound	Upper Bound
(Constant)	− 11.93	2.92		− 4.09	< 0.001*	− 17.73	− 6.14
Concha bullosa	2.10	0.79	0.19	2.65	0.01*	0.52	3.67
HT middle width	2.28	0.44	0.42	5.16	< 0.001*	1.40	3.16

Dependent Variable: SDA

F(3, 99) = 43.94, *P* < 0.001, R^2^ = 0.76, *p < 0.05 Statistically Significant, *p* > 0.05 Non Significant, NS

Ordinal logistic regression revealed that width of the middle HT (*p* = 0.01) and presence of concha (*p* = 0.04) had a significant influence on the type of NSD (Table 12).

**Table 12:** Ordinal Logistic regression to assess the influence of study variables on type of NSD

		Estimate	Std. Error	Wald	df	*p*-value	95% Confidence Interval		Odds ratio	95% Confidence Interval of odds ratio	
							Lower Bound	Upper Bound		Lower Bound	Upper Bound
Threshold	[Type = 1]	14.01	3.12	20.10	1	< 0.001*	7.89	20.13			
	[Type = 2]	15.93	3.23	24.26	1	< 0.001*	9.59	22.27			
Variables	Age	0.03	0.02	2.90	1	0.09 (NS)	− 0.01	0.07	1.03	1.00	1.07
	HT – Anterior Width	− 0.01	0.57	0.00	1	0.06 (NS)	− 1.13	1.12	0.99	0.32	3.05
	HT – Middle Width	1.58	0.64	6.13	1	0.01*	0.33	2.84	4.87	1.39	17.10
	HT – Posterior Width	− 0.23	0.40	0.34	1	0.07 (NS)	− 1.02	0.55	0.79	0.36	1.74
	NHT – Anterior Width	− 0.08	0.32	0.06	1	0.81 (NS)	− 0.71	0.55	0.93	0.49	1.73
	NHT – Middle Width	0.45	0.34	1.80	1	0.18 (NS)	− 0.21	1.11	1.57	0.81	3.05
	NHT – Posterior Width	− 0.21	0.22	0.89	1	0.35 (NS)	− 0.64	0.22	0.81	0.53	1.25
	[Concha Bullosa-present]	− 0.94	0.51	3.35	1	0.04*	− 1.95	0.07	0.39	0.14	1.07
	[Concha Bullosa-absent]	0			0						
	[Gender-male]	0.44	0.48	0.82	1	0.37 (NS)	− 0.51	1.39	1.55	0.60	4.00
	[Gender-female]	0			0						

Model Chi square (9) = 78.76, *p* < 0.001*

Cox and Snell R^2^ = 0.545, Nagelkerke R^2^ = 0.616

^*^p < 0.05 Statistically Significant, *p* > 0.05 Non Significant, NS

## Discussion

Nasal turbinate hypertrophy can occur due to congenital or acquired causes. Acquired causes can be secondary to conditions like allergic rhinitis, dust allergy, pregnancy, hyperthyroidism, and rebound nasal congestion due to extended use of topical decongestants [13]. Although turbinate hypertrophy is transient, the presence of NSD makes the hypertrophy persistent [14]. NSD is a common problem in the middle eastern population [11]. Computed Tomography (CT) is currently being used to study NSD and associated sino-nasal conditions [15]. Since inflammatory sinus diseases are often recurring and require repetitive imaging CBCT could be a suitable alternative to CT since the former is associated with a significantly lower dose of radiation [16]. However, a recent survey revealed that the knowledge regarding applications and advantages of CBCT is very limited among otolaryngologists [17]. This study was conducted to analyze NSD, CB, and associated changes in the inferior nasal turbinate using CBCT.

In the present study, the mean SDA of the patients with NSD was 12.03 degrees. Similar SDA values were reported by Demir D et al., Orhan I et al., Keles B et al. and Serifoglu I et al. in their studies conducted on the Turkish population [5, 7, 18, 19]. A study conducted in Serbian population also reported a similar mean SDA value, as reported in the present study [20]. Another study conducted using CT on the Iraqi population also reported mean SDA values as reported in the present study [21]. However, in a study by conducted by Codari M et al. in the Italian population, the mean SDA was 17.3 degrees [22]. Another CT-based study conducted in the Iranian population also reported of lower mean SDA values compared to the present study [23].

In the present study, NSD was classified as mild, moderate, and severe based on SDA. In a recent study, few authors have categorized SDA from Type I to Type IV and reported that Type II SDA (5–10 degrees) was the most common in occurrence [24]. Although a majority of the studies have reported mean SDA values similar to that of our present study, there are some studies that have reported different mean SDA values. This difference in mean SDA values among studies could be attributed to racial variations in the study population and the measurement techniques used in different studies [20].

In the present study, the occurrence of the types of NSD was not correlated to age of the study subjects. Similar results were presented by Goergen MJ et al. and Yonis MA et al. in the American and Egyptian populations [25, 26]. Besides the occurrence of the types of NSD did not show any difference between the genders of the patients. This finding was consistent with the study results of Serifoglu I et al., Altunay ZO et al. Shobeiri et al. [19, 27, 28]. On contrary a recent Turkish study reported a significantly high mean SDA among men compared to women [29].

Our study results are highly consistent with the literature evidence that there is no notable difference in the mean SDA concerning the age and sex of study subjects. In the current study the mean width of NHT and the mean width HT in patients with NSD was 7.27 ± 0.67 m and 10.46 ± 2.63 mm respectively. Similar measurements were presented in many CT studies from Egypt and Iraq [30–33].

A significant difference was noted between the anterior, middle, and posterior widths of HT and NHT in our study. A similar difference was published by many authors in patients with NSD [6, 7, 34, 35]. However, the current study used CBCT while all the previous studies were carried out using CT.

Various reference points were used by different authors for the measurement of turbinate width. Orhan et al. measured the widest part of the turbinate while Kang JW et al. measured it at the front part of the vertical portion of the uncinated process and Tombilson CM et al. used ostium as a standard point for measurement [7, 35, 36].

While most of the studies used a single reference point for width measurement Egeli et al. used three standard reference points [6]. In the present study turbinate width was measured at 3 standard points. Measurement of width at multiple standard points is important because the turbinates show significant changes in width in different areas [8].

It was initially assumed that the compensatory hypertrophy of the nasal turbinates was purely mucosal [3]. Later radiographic and histopathological studies revealed that the hypertrophy of the bony component of the turbinate along with the mucosal component [36].

Further studies revealed that the bony component of the inferior turbinate becomes thicker, spongier, and tends to arch medially into the nasal airway [3]. The mucosal component undergoes hypertrophy by building up a rich network of venous sinusoids and thereby acquire an exaggerated capacity to expand [3]. Researchers have also detected histopathological differences in the NSD induced compensatory and allergic hypertrophy of the nasal turbinate [37]. Compensatory hypertrophy of the inferior turbinate is usually associated with increased venous sinusoids, fibrosis around the blood vessels and normal glands, whereas allergy-induced hypertrophy comprises glandular hyperplasia and significant edema [37, 38].

The submucosal blood vessels and glands of the inferior turbinates are under autonomic control. They a vital role in maintaining the local homeostasis by controlling the nasal secretions, nasal patency, and humidification of air in the nasal cavity [39]. In compensatory hypertrophy, this delicate homeostasis is altered [40].

In the present study, there was a significant difference in the anterior, middle, posterior and mean width of HT among mild, moderate, and severe types of NSD. Similar findings were observed in the CT-based studies by Orhan et al., Tombilson et al. and Egeli et al. [6, 7, 34]. However, Demir et al. report no significant difference in the width of the turbinates with alteration in NSD. [1]. This finding could be attributed to the fact that Demir et al. evaluated middle turbinate hypertrophy whereas inferior turbinate hypertrophy was evaluated in the present study.

Two theories are proposed to describe the correlation between turbinate hypertrophy and NSD [6]. The first and widely accepted theory states that the hypertrophy of the contralateral inferior turbinate occurs as a compensatory reaction to NSD. The second theory states that the unilateral growth of the nasal turbinate results from genetic causes or early life trauma which is likely to exert pressure on the growing nasal septum throughout childhood and puberty. This constant pressure leads to the deviation of the nasal septum to the contralateral side. The theory considerably substantiates the fact that bony enlargement significantly contributes to the measured thickness of the turbinate in compensatory hypertrophy [6].

In the present study, there was a significant increase in width of HT, NHT, and the degree of SDA when CB was present. Similar findings were reported by Tombilson et al. and Stallman et al. [9, 35]. The literature suggests a strong association between NSD and CB however, their relationship is not clear in terms of cause–effect relationship [35]. The theory of “e vacuo hypothesis” states that CB only fills the space created by NSD [39].

Studies have also suggested that the septum is typically pushed away from the CB when present unilaterally and pushed away from the dominant concha bullosa when present bilaterally when bilateral [9, 38].

In the present study, ordinal logistic regression revealed that width of the middle HT and presence of concha (*p* = 0.04) had a significant influence on the type of NSD which is similar to findings in the CT-based study by Tombilson CM et al., that showed that CB had a significant influence on the degree of NSD [35]. In our study, the middle width of the HT had a significant influence on the NSD. Mrig et al. found that the medial mucosa undergoes maximal hypertrophy compared to the rest of the areas [8]. This might be the probable reason for the significant association between the middle width of HT and NSD observed in our study.

NSD associated compensatory hypertrophy leads to the chronic nasal obstruction which can significantly affect the quality of life, productivity, and finances [40]. When drug therapy is unsuccessful in reducing compensatory hypertrophy, a surgical line of management is often recommended [41, 42]. A recent expert consensus meets states that inferior turbinoplasty is an effective adjunctive procedure to septoplasty in the presence of hypertrophic inferior turbinates [43, 44]. The diagnosis, treatment and follow-up of compensatory hypertrophy associated with NSD would require multiple imaging studies. Although CT is used for imaging for compensatory hypertrophy associated with NSD, CBCT could be used a suitable alternative with a relatively lower dose of radiation [45].

## Conclusion

From the results of the study we can conclude that the middle width of the inferior hypertrophic turbinate and the presence of concha influence the degree of NSD. The present study results also recommend the use of CBCT as a substitutive low radiation dose imaging modality for evaluation of NSD, CB, and associated inferior turbinate hypertrophy.

## Data Availability

The corresponding can be contacted for raw data. The data is also available at figshare; https://doi.org/10.6084/m9.figshare.14413730

## Abbreviations

CT: Computed tomography
CBCT: Cone beam computed tomography
CB: Concha bullosa
NSD: Nasal septal deviation
SDA: Septal deviation angle
HT: Hypertrophied turbinate
NHT: Non-Hypertrophied turbinate
